# Continuous Glucose Monitoring Sensors: Past, Present and Future Algorithmic Challenges

**DOI:** 10.3390/s16122093

**Published:** 2016-12-09

**Authors:** Andrea Facchinetti

**Affiliations:** Department of Information Engineering, University of Padova, Padova 35131, Italy; facchine@dei.unipd.it; Tel.: +39-049-827-7669

**Keywords:** signal processing, diabetes, calibration, model identification, filtering, prediction

## Abstract

Continuous glucose monitoring (CGM) sensors are portable devices that allow measuring and visualizing the glucose concentration in real time almost continuously for several days and are provided with hypo/hyperglycemic alerts and glucose trend information. CGM sensors have revolutionized Type 1 diabetes (T1D) management, improving glucose control when used adjunctively to self-monitoring blood glucose systems. Furthermore, CGM devices have stimulated the development of applications that were impossible to create without a continuous-time glucose signal, e.g., real-time predictive alerts of hypo/hyperglycemic episodes based on the prediction of future glucose concentration, automatic basal insulin attenuation methods for hypoglycemia prevention, and the artificial pancreas. However, CGM sensors’ lack of accuracy and reliability limited their usability in the clinical practice, calling upon the academic community for the development of suitable signal processing methods to improve CGM performance. The aim of this paper is to review the past and present algorithmic challenges of CGM sensors, to show how they have been tackled by our research group, and to identify the possible future ones.

## 1. Introduction

Continuous glucose monitoring (CGM) sensors are portable devices that allow measuring and visualizing the glucose concentration almost continuously (usually every 1–5 min) for several days (so far up to seven days). CGM sensors are composed of three main elements: (i) a needle-based sensor, which is usually inserted in the abdominal subcutis and measures an electrical signal proportional to the glucose concentration present in the interstitial fluid; (ii) a transmitter, which is applied over the sensor and is aimed at transmitting the signal; and (iii) a portable device, which receives the signal and visualizes it on a monitor. In particular, the final signal is a glucose concentration, and it is obtained by converting the raw electrical signal measured by the sensor via a calibration procedure that exploits one or more reference blood glucose (BG) values collected using a portable self-monitoring BG (SMBG) device. Note that the calibration can take place either in the transmitter or in the receiver. Furthermore, because of the skin-sensor interactions and possible variability of sensor sensitivity over time, CGM sensors require re-calibration every 12 h.

The first CGM sensor was introduced more than 15 years ago and was intended as retrospective instrument to monitor the glucose concentration for 72 consecutive hours with the objective of improving the knowledge about glucose fluctuations and dynamics, revealing hypoglycemic and hyperglycemic episodes that were impossible to detect using sparse SMBG measurements [1]. Since then, CGM sensors have been constantly improved by adding new features: the real-time visualization of glucose concentration value and its trend [2], the generation of acoustic and visual alerts when settable hypoglycemic and hyperglycemic thresholds are overcome [3], the increase in sensor duration [4], and reduction of hardware size [5], until it is possible to be connected directly to smart-phones and access glucose concentration data via remote monitoring [6].

The availability of a quasi-continuous time signal of glucose concentration represented a revolution in glucose monitoring. From a clinical point of view, it has been widely demonstrated that the additional information provided by CGM sensors, when used in conjunction with SMBG data, improves the quality of glucose control [7,8]. From an academic point of view, the availability of CGM data stimulated, over the last 15 years, the development of several CGM-based applications, e.g., algorithms for the prediction of future glucose concentration to generate preventive hypo/hyperglycemic alerts [9,10,11,12,13], for the real-time modulation of the basal insulin administration [14,15,16], and for the detection of faults with glucose sensor–insulin pumps system [17,18,19,20,21]. Even more interesting is that CGM sensors enabled the realization of the artificial pancreas (AP), i.e., a device designed mainly for Type 1 diabetes (T1D), which is aimed at maintaining the BG concentration within the safety range by automatically injecting insulin via an insulin pump controlled by a closed-loop control algorithm [22,23,24,25].

All of the aforementioned applications and uses of CGM sensors strongly rely on the assumption that the information provided by CGM devices is accurate and reliable. However, as largely discussed in Sparacino et al. [26], CGM sensors could be affected by lack of accuracy and reliability, which can negatively condition the performance of CGM-based applications and systems and limit their porting in clinical practice. For this reason, improving the performance of CGM sensors and the reliability of CGM data via suitable signal processing methods was identified as a key issue to be addressed.

The aim of this paper is to review the past and present algorithmic challenges of CGM sensors, in order to to present how they have been tackled, with particular focus on the work done by our research group, and identify what we envision will be the possible future versions of CGM sensors.

## 2. The Past: The “Smart” CGM Sensor

**Figure 1:**
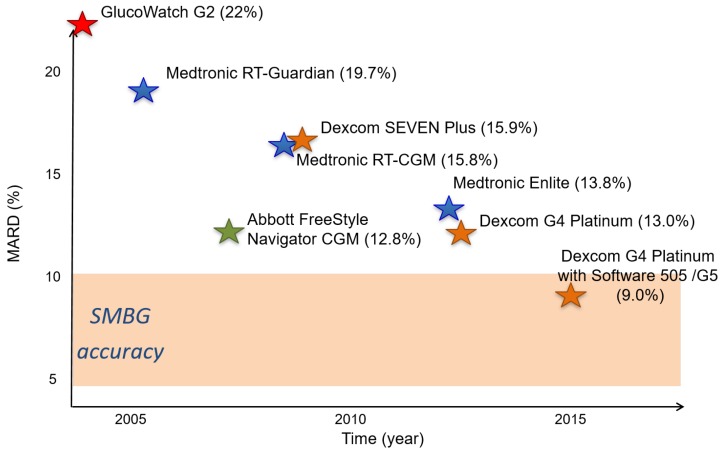
The accuracy timeline of CGM sensors over the last 15 years.

As reviewed in Sparacino et al. [26], the main past challenges were related to the improvement of the quality of the data coming from CGM devices via suitable signal processing methods. In fact, even if CGM sensors were considered to be revolutionary devices for glucose monitoring, the limited accuracy and reliability of CGM data represented a bottleneck for both the daily use of CGM in the clinical practice and the development of CGM-based applications. The evolution over the last 15 years of CGM accuracy, calculated as mean absolute relative deviation (MARD) with respect to very precise and accurate BG reference readings, of some of the most important commercial CGM sensors, is shown in Figure 1. As is visible, at the time of writing the work [26], the accuracy of commercial CGM sensors was significantly higher than the nominal SMBG systems. In particular, the following three main areas were identified as the keys to enhancing CGM sensor performance: (i) improving the precision of CGM data, i.e., reducing the random noise component overlapped to the true glycemic signal; (ii) improving the accuracy, reduction or even elimination of the systematic differences (i.e., biases and time-drifts) observable between CGM data and gold-standard BG measurements due, e.g., to imperfect sensor calibration or variability in time of sensor sensitivity; and (iii) improving the timeliness of CGM hypo/hyperglycemic alerts by predicting the future BG concentration and generating the prevention of hypo/hyperglycemic alerts.

These three key challenges have been overcome by the creation of the algorithmically “smart” CGM sensor, which consists of placing, in a cascade of the output of a commercial CGM sensor, three software modules for (i) denoising; (ii) enhancement; and (iii) prediction [27,28]. The denoising module is aimed at reducing the uncertainty due to measurement noise on CGM data by exploiting real-time digital filters. The goal of the enhancement module is improving the accuracy of CGM data by reducing systematic differences between reference BG measurements and CGM data due to, for example, BG-to-interstitial glucose (IG) kinetics and/or variability in time of CGM sensor sensitivity. Finally, the prediction module is aimed at mitigating the occurrence of hypo/hyperglycemic events by the generation of preventive alarms when the short-term prediction of future glucose concentration exceeded hypo/hyperglycemic thresholds.

**Figure 2:**
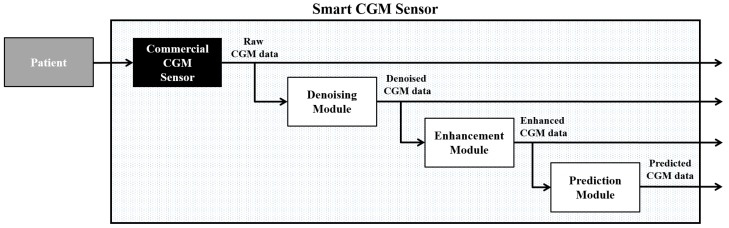
The smart CGM sensor architecture, which consists of placing, in cascade to the output of a commercial CGM sensor, three software modules, able to work in real time, for denoising the random noise component, enhancing the accuracy, and predicting the future glucose concentration (adapted from [27]).

The smart CGM sensor architecture is shown in Figure 2.

The “smart” CGM sensor architecture is flexible, i.e., any kind of algorithm working in real time and satisfying the requirements of the specific module can be used.

In the implementation, we proposed in Facchinetti et al. [27], that the denoising module contained an adaptive self-tunable Bayesian smoother [29,30] able to automatically estimate in real time the signal-to-noise ratio present on the CGM data and able to perform an optimal and computationally efficient real-time smoothing thanks to a Kalman filter-based implementation. Other denoising algorithms that can be used here are those, e.g., of Chase et al. [31], Palerm and Bequette [32], Kuure-Kinsey et al. [33], and Mahmoudi et al. [34].

With regard to the enhancement module, we proposed a stochastic deconvolution-based re-calibration algorithm [35], which re-scales the CGM data using a simple linear regressor whose parameters are re-calculated every time a new SMBG value is available. The key feature of this algorithm was to use stochastic regularized deconvolution of CGM trace to create a continuous BG profile before calculating the regression function parameters to compensate for the presence of BG-to-IG dynamics. Recently, the algorithm has been further improved by adding priors in the parameters’ estimation step [36]. Other enhancement/recalibration algorithms that can be exploited in this module are, e.g., those of Barcelo-Rico et al. [37,38], Mahmoudi et al. [34], and Kirchsteiger et al. [39].

Finally, the algorithm we employed in the prediction module is a simple but effective predictor based on an autoregressive model of order one [40], whose key feature is the real-time estimation of the predictor’s parameters using a recursive least squares implementation, exploiting a forgetting factor to smartly weight previously acquired data. It is worth noting that more sophisticated prediction algorithms, also exploiting other signals like the amount of insulin injected or physical activity, can be employed, e.g., those of Zhao et al. [9,12], Zecchin et al. [13,41], Turksoy et al. [10], Zarkogianni et al. [11], and Georga et al. [42,43].

The algorithmically “smart” CGM sensor concept has been validated on a dataset consisting of 23 T1D individuals whose glucose concentrations were monitored using both the SEVEN Plus CGM sensor (Dexcom Inc., San Diego, CA, USA) and frequent gold-standard BG value [27]. Results showed that, thanks to the employment of signal processing algorithms only, it was possible to reduce the MARD of the Dexcom SEVEN Plus CGM device from 15.1% to 10.3%. This result was very important since it showed that the accuracy of CGM sensors could be lowered to values close to those observed for SMBG systems.

These results stimulated Dexcom Inc., one of the leading manufacturers of CGM devices, to adopt the “smart” CGM architecture to improve the accuracy of their new devices. In particular, thanks to the collaboration between the University of Padova and Dexcom Inc., Dexcom Inc. re-designed part of the signal processing code employed in the Dexcom G4 Platinum sensor by taking inspiration especially from the denoising and enhancement algorithms employed in the “smart” CGM sensor architecture [27]. The new signal processing code was released by Dexcom Inc. under the name of “software 505”, which allowed by itself (i.e., without changing any hardware component) to reduce the MARD of the G4 Platinum from 13% to 9% [5,44,45], making this, in 2014, device the first CGM sensor reaching one-digit accuracy.

## 3. The Present: The Nonadjunctive CGM Use

In the previous section, we showed how the main challenges highlighted in the work of Sparacino et al. [26] were tackled by our research group with the creation of the “smart” CGM sensor architecture. However, despite the fact that the accuracy of CGM sensors reached levels comparable to that of commercial SMBG devices, CGM sensors remain approved only for an “adjunctive” use, i.e., no therapeutic decisions (e.g., insulin dosing) can be taken on the basis of CGM data (a confirmatory SMBG value is always required). From a regulatory point of view, SMBG systems remain the only portable glucose measurement system approved by regulatory agencies for insulin dosing (the only exception being the Dexcom G5 Mobile system in the European Union).

It is becoming a common perception that CGM sensors are accurate enough to be used for therapeutic decisions [46] and the academic research is now focused on this new challenge, i.e., demonstrating both the safety and efficacy of CGM sensors for the nonadjunctive use. Only Kovatchev et al. [47] explicitly addressed this problem and tried to give some indications on this topic. By modifying retrospectively some CGM traces using the so-called “net-effect” model [48], the authors reached the preliminary conclusion that CGM sensors with MARD ≤10% could be suitable for insulin dosing decisions. The work also highlighted that, in order to draw more solid conclusions, additional investigation is needed, and, in particular, the focus should be on that part of CGM sensors presenting a bad accuracy (i.e., those with high MARD).

On the basis of the results of Kovatchev et al. [47], proving the safety and efficacy of the nonadjunctive use of CGM sensors becomes a typical problem in which it is important to test and understand what happens in that small part of the population whose behavior is different from the average, i.e., in the case of rare but not so rare events [49]. There are three main strategies that, in theory, could be used to investigate such a population: (i) the retrospective analysis of already acquired CGM data; (ii) in vivo clinical trials; and (iii) in silico clinical trials (ISCT). The retrospective analysis and modification of CGM data is not suitable for the scope. In fact, the main limitation is the impossibility of correctly changing the already acquired data to understand what would have happened if the insulin dosing was based on CGM rather than SMBG data [50]. The most logical solution would be an in vivo clinical trial. However, this option would also not be the most suitable, since the number of patients required to ensure the exploration of all extreme cases and to test the efficacy on the whole T1D population would be very large (thousands).

The solution to overcome the limitations of the two previous approaches is the use of ISCT. The advantages of ISCT are several: (i) low cost and low time for realization; (ii) possibility of generating thousands of virtual patients; (iii) possibility of testing extreme cases (e.g., investigating insulin dosing using CGM sensors with bad accuracy); and, maybe the most important, (iv) the possibility of testing the effect of using device A in place of device B in the same identical scenario, which means, in our case, testing what would have happened by using CGM in place of SMBG data for insulin dosing. However, to generate reliable results from ISCT, a large-scale simulation model is required that is able to describe both the physiological variability present in the population under analysis and the variability observed on the technology behavior [49].

To prove the safety and effectiveness of the nonadjunctive use of CGM sensors, our research group is developing the T1D decision-making (T1D-DM) model, i.e., a model of T1D patients making SMBG-based or CGM-based treatment decisions, like insulin dosing or CHO intake [51].

**Figure 3:**
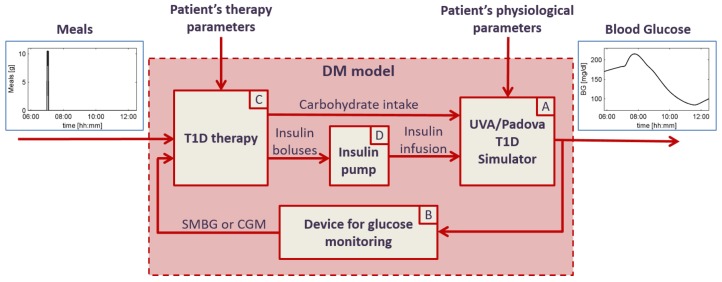
The T1D-DM model developed to generate ISCT to test SMBG-based or CGM-based treatment decisions.

The T1D-DM model is a mathematical model of T1D patients making treatment decisions, and it is graphically represented in Figure 3. The T1D-DM receives for input the pattern of the meals defined in the scenario to be simulated and patient-specific parameters describing both patient’s physiology and behavior. In particular, the behavior is characterized by all the variables describing how the patient monitors the glucose concentration (e.g., timing, frequency, etc.) and how/when he/she makes treatment decisions. The model output is the patient’s BG concentration that is simulated every minute, thus resulting in a quasi-continuous time profile. The T1D-DM model includes four main components:

(A) *the UVA/Padova T1D simulator*, which receives for input the physiological parameters of the specific virtual patient, the CHO intake and insulin infusion, and outputs the BG concentration profile. The UVA/Padova T1D simulator is a large-scale maximal computer model of glucose, insulin and glucagon dynamics in patients with T1D, described by 13 differential equations and provided with three different populations: 100 adult, 100 pediatric and 100 adolescent virtual subjects [52,53]. Each virtual subject is characterized by 36 physiological parameters, able to describe the inter-individual variability observed in the T1D population. The UVA/Padova T1D simulator was originally designed to generate single-meal scenarios and was accepted by the U.S. Food and Drug Administration (FDA) to substitute the pre-clinical for certain insulin treatments in 2008. Recently, thanks to the development and embedding of inter- and intra-day variability of the insulin sensitivity, the new version of the UVA/Padova T1D simulator allows for generating physiological glucose profiles of T1D on multiple-day scenarios [54,55];
(B) *models of SMBG and CGM devices*, able to reliably reproduce all the technological variability (in terms of accuracy) that can be observed in real life. These models receive for input the BG concentration from the UVA/Padova T1D simulator and outputs the CGM and SMBG values, respectively. The SMBG measurement error is sampled from a composite distribution obtained combining a skew-normal density function, to describe the central part of the distribution, the exponential functions, and the tails [56]. The CGM sensor model is able to describe all of the key components of the sensor error, i.e., the variability of the time delay due to the BG-to-IG diffusion process, the variability of the calibration error, and the variability of the random noise component by sampling from appropriate distributions as described in depth in [57,58,59];
(C) *a model of treatment rules and subject behavior*, able to reproduce all the variability in the habits of the diabetic patients and in their use of BG monitoring technologies [51]. Specifically, this module simulates the patient behavior in using SMBG and/or CGM information to make treatment decisions like tuning meal insulin doses and triggering correction boluses and CHO hypo rescues. The inputs of the model the SMBG and/or the CGM measurements (which includes not only the glucose readings, but also trend arrows, alerts and alarms), the meal scheduling and patient’s therapy parameters, like the CHO-to-insulin ratio (CR) and the correction factor (CF). The model outputs are insulin boluses, obtained as the sum of meal boluses and correction boluses, and CHO intake, obtained as the sum of meals’ CHO and hypotreatments;
(D) *the insulin pump model*, which is simply an actuator receiving for input the dose of insulin boluses and outputting the insulin delivery pattern containing the minute by minute dose of insulin injected [51].

The preliminary application of the T1D-DM model to test the nonadjunctive use of CGM sensors was published by our group in Vettoretti et al. [51]. In this work, the T1D-DM model was used to generate a seven-day ISCT on 20 adults, with three meals per day with variability in time, amount and meal bolus behavior. Each virtual patient underwent the same ISCT twice: the first time, the insulin dosing was based on SMBG measurements, whereas the second time, it was based on CGM data. The results of standard outcome metrics, e.g., time in severe hypo, time in hypo, time in target, time in hyper, time in severe hyper, and the number of hypo/hyperglycemic events, calculated for both CGM and SMBG scenarios, supported the non-inferiority of CGM vs. SMBG.

Very recently, the T1D-DM model was used in collaboration with Dexcom Inc. (San Diego, CA, USA) to run simulations to assess safety and effectiveness of the nonadjunctive use of the Dexcom G5 Mobile sensor. In particular, a two-week ISCT was generated on 40,000 virtual unique adult and pediatric patients, showing that, in both adult and pediatric populations, the risk of hypo and hyperglycemia using CGM for insulin dosing was equivalent or even lower than that of using SMBG. These results were included among all the material (clinical study data, analysis of human factors, opinion of clinicians and experts, and the testimony of CGM sensor users) that Dexcom Inc. presented to the Clinical Chemistry and Clinical Toxicology Devices Panel of the U.S. FDA’s Medical Device Advisory Committee on 21 July 2016 to ask for a change of label for the G5 Mobile device from adjunctive to nonadjunctive use [60,61].

## 4. The Future: New Challenges

In the previous sections, it has been shown that the accuracy of CGM sensors is no longer a bottleneck for CGM-based applications thanks to “smart” signal processing algorithms, and that CGM sensors are safe and effective for the nonadjunctive, even if the final official approval by the U.S. FDA is still pending. The new challenges are now more related to improving the usability of CGM sensors and exploiting the large amount of data coming from CGM devices.

Two important research topics are directly connected with the nonadjunctive use of CGM sensors. The first is the definition of the optimal rules for dosing insulin on the basis of CGM data. In fact, the rules available so far, e.g., those for the calculation of the insulin bolus at meal time, are designed for SMBG devices and not to exploit the continuous-time information that CGM data is able to provide. For instance, in the standard T1D basal+bolus insulin therapy administered via insulin pumps, the amount of insulin to be injected as a bolus at meal-time $I_{B}$ is calculated as follows:

(1) $$I_{B}=\left(\frac{CHO}{CR}+\frac{BG_{mt}-BG_{target}}{CF}\right),$$

where CF is the correction factor, CR is the CHO-to-insulin ratio, $BG_{mt}$ is the BG concentration at meal time measured via SMBG, and $BG_{target}$ is the target BG value that the T1D patient would like to reach after the meal. However, CGM sensors carry more information than a simple point-of-care BG value measured by SMBG, e.g., trends, past history of hypo- and hyperglycemia, etc. This additional info should be exploited to modify the current rules for insulin dosing or to design new ones to further improve the glucose control during and after the meal. For instance, the bolus at meal-time $I_{B}$ could be modified to take into account the glucose trend information, e.g., by increasing the amount of insulin if the trend is positive and lowering it if negative, as follows:

(2) $$I_{B}=\left(\frac{CHO}{CR}+\frac{CGM_{mt}-BG_{target}}{CF}\right)\left(1+CGMtrend_{mt}*\frac{P}{100}\right),$$

where $CGM_{mt}$ is the CGM value at meal time, $CGMtrend_{mt}$ is the CGM trend at meal time (e.g., expressed as mg/dL/min) and *P* is a percentage value (0≤P≤100). In addition, ISCT represents here the best option for a deep understanding of whether these modifications could be effective or not. In particular, our research group is testing in silico the effectiveness of the modification proposed in Equation (2) using the T1D-DM in a single-meal scenario. The preliminary results on 30 virtual T1D patients suggest that the optimal percentage *P* for the modulation is 30% when the trend is decreasing and 40% when the trend is increasing [51].

The second important research topic directly connected to the nonadjunctive use of CGM sensors is the reduction of the number of calibrations. In fact, even if CGM sensors are close to being approved for making therapeutic decisions like insulin dosing, i.e., confirmatory SMBGs being no longer required, SMBG values are still needed at least twice a day to calibrate CGM sensors. Therefore, reducing the number of calibrations, or even eliminating them, is key to improving the daily usability of the CGM device and effectively reducing the technological burden for T1D patients, who still have to carry the SMBG system with them for CGM calibration. The reduction/elimination of calibrations in CGM systems is further pressured by the recent advent of the Abbott Freestyle Libre (Abbott Diabetes Care, Alameda, CA, USA) [62,63]. The Libre system is labeled as a device for the flash monitoring of glucose concentration, i.e., to be used in substitution of SMBGs, since it provides point-of-care glucose concentration values when the portable receiver is passed over the sensor. However, the sensor measures the glucose concentration continuously, like CGM devices, but with the key features of lasting for 14 days and requiring no calibrations, suggesting that improvements in CGM are possible. To reduce/eliminate the calibrations in CGM systems, the use of new calibration functions and suitable priors for calibration parameters is giving promising results. In a recent study of our group [64], we demonstrated that the number of calibrations can be reduced from two to one per day thanks to the use of the new calibrations functions that allow taking into account the variability in time of sensor sensitivity and ad hoc priors that improve the real-time estimation of the calibration function parameters.

Another research area that requires attention is fault detection. In fact, the availability of a quasi-continuous time BG profile could help in detecting transient and permanent faults of the CGM sensor-insulin pump system, e.g., compression artifacts due to external pressure on the sensor that can cause a rapid non-physiological lowering of the BG concentration, or failures of the infusion set of the insulin pump. These algorithms are particularly important for AP systems [65], where a prompt detection of the failures of both the CGM sensor and the insulin pump is key for the safety of the patient, e.g., avoiding an incorrect or suboptimal calculation of the insulin dosage to be injected by the closed-loop control system. Some algorithms for fault detection have been presented in the literature [17,18,19,20,21], but they are still prototypes, and more investigation is required on their effectiveness before porting them into AP systems.

## 5. Conclusions

CGM sensors have revolutionized the real-time monitoring of glucose concentration, enabling applications that, before their advent, were impossible to realize, like AP systems. In this paper, we reviewed the status of the algorithmic challenges of CGM systems and presented in detail how our research group handled them. We have shown how the algorithmic open issues identified by Sparacino et al. [26] have been tackled by the creation of the “smart” CGM sensors, which allowed improving precision, accuracy and timeliness of hypo/hyperglycemic alerts of commercial CGM sensors. The improvement of CGM sensor performance to a level similar to that of SMBG devices has recently stimulated research on the possibility of using CGM for therapeutic decisions, i.e., nonadjunctively. In particular, we have shown how suitable ISCT generated using realistic large-scale simulation models could be used to prove safety and effectiveness of the nonadjunctive use of CGM sensors. Finally, we have highlighted some of what we envision will be the most important future algorithmic challenges, i.e., the necessity to modify or change the current rules for insulin dosing to take into account the information carried by CGM sensors, the need of reducing or even eliminating CGM sensor calibrations, and the importance of exploiting CGM data in conjunction with other continuous-time signals (like insulin pump data) to detect failures of the CGM sensor-insulin pump system.

## Abbreviations

The following abbreviations are used in this manuscript:

AP	artificial pancreas
BG	blood glucose
CF	correcton factor
CR	carbohydrate-to-insulin ratio
CGM	continuous glucose monitoring
CHO	carbohydrate
IG	interstitial glucose
ISCT	in silico clinical trial
MARD	mean absolute relative difference
SMBG	self-monitoring blood glucose
T1D	Type 1 diabetes
T1D-DM	Type 1 diabetes-decision making
UVA	University of Virginia
